# Management work mode of college students based on emotional management and incentives

**DOI:** 10.3389/fpsyg.2022.963122

**Published:** 2022-07-28

**Authors:** Xiang Ding

**Affiliations:** ZWU Faculty of Design and Architecture, Zhejiang Wan li University, Ningbo, China

**Keywords:** college student management, emotional management and motivation, speech emotion recognition, artificial neural networks, emotional management of college students

## Abstract

The student management work model in colleges and universities is an effective plan for college student management, but the traditional college student management work is not very good in terms of student psychology, resulting in negative attitudes such as low learning desire, low learning efficiency, and inactive learning. In recent years, with the development of artificial intelligence technologies such as sentiment analysis and incentive theory, emotional management and incentive theory have been applied to the management of college students. The emotional management and incentive model is a way to help college students get rid of psychological obstacles and guide students to establish positive and correct values by predict and analyze the psychological state of college students through language emotion recognition and BP neural network. This paper compares the college student management work model based on emotional management and incentives with the traditional college management work mode through experiments. The results show that the students’ learning enthusiasm is better than the traditional college student management work mode based on emotional management and incentives. The student management work model in colleges and universities is 15.8% better, and the students’ grades have improved by 12.5%; the college student management work model based on emotional management and incentives also has a positive role in helping students’ mental health. The way of emotional management and motivation can make better use of college students’ psychology to effectively manage students and guide students to develop in a good direction.

## Introduction

The development of education has made more and more students in colleges and universities, but at the same time, college students have also encountered many problems in the college student management model, such as not active in class, lack of learning goals, and failure to plan college life well. Therefore, in recent years, more attention has been paid to the management of college students. The traditional management work in colleges and universities focuses on students’ diet, daily life, study, etc and pays little or no attention to the psychological level of students. With the emphasis on the psychology of college students, the traditional college management work is gradually eliminated. Contemporary college students are active in thinking and pursuing freedom in their hearts. In order to better manage college students, a management model of emotional management and incentives should be used and live. Emotional management is a process of identifying and analyzing students’ emotions and making corresponding management strategies. Incentive mode is a process of using external media to guide students to make positive responses. Use emotional management and incentive management mode to guide students’ psychology and cultivate college students’ excellent qualities such as positiveness, self-discipline, and hard work. Therefore, this paper has research significance.

College students are the mainstay of the current era, and the management of college students is becoming more and more important. Many researchers are devoted to the management of college students. Among them, Yu proposed an evaluation method to evaluate and manage college students by analyzing the management status of college students (Yu and Huo, 2017). Kaviyarasi uses data mining and other technologies to obtain college student information, and formulates corresponding college student management plans based on college student information (Kaviyarasi and Balasubramanian, 2018). Wang’s research pointed out that the student management model that obtained student information with big data and constructed can manage college students well (Wang et al., 2021). Wang builds a management model for college students’ sports problems, which can effectively manage the sports aspects of college students (Wang, 2017). Alan analyzes the daily life of students by establishing a university student management model for better management (Alan et al., 2018). The establishment of management model and the acquisition of student information can effectively manage college students, but there is no research on the psychological level of students.

The effective management of college students requires not only the management of students’ life but also the psychological management. Related researches combine emotional management with the way of motivation and the management mode of college students. Among them, Sri makes corresponding management plans by analyzing the emotions of college students in class (Sri, 2020). Zhai uses emotion recognition analysis technology to help lecturers understand the psychology of college students, which can improve students’ learning efficiency (Zhai et al., 2020). Nandwani judges the emotions of each college student through emotion detection, and guides students to learn in a motivating way (Nandwani and Verma, 2021). Yu researches the positive and negative emotions of college students, and formulates college management methods based on students’ emotional characteristics to provide students with a better life in school (Yu et al., 2018). Sun’s research found that incentive-based college teaching can improve the quality of college teaching (Sun, 2017). Emotional management and motivational college student management can analyze students’ emotions well and help students improve their learning efficiency, but the method of emotional management is not optimal.

This paper combines emotional management and motivation to conduct emotional analysis and motivation guidance for college students, and analyzes the advantages and disadvantages of the two management modes by comparing with the traditional management mode of college students. The innovations of this paper are as follows: (1) to analyze and manage college students by combining emotional management and incentives and (2) comparing with the traditional management mode of college students, it highlights the management advantages of combining emotional management and incentives.

## Emotional management and incentive management methods for college students

**Figure 1:**
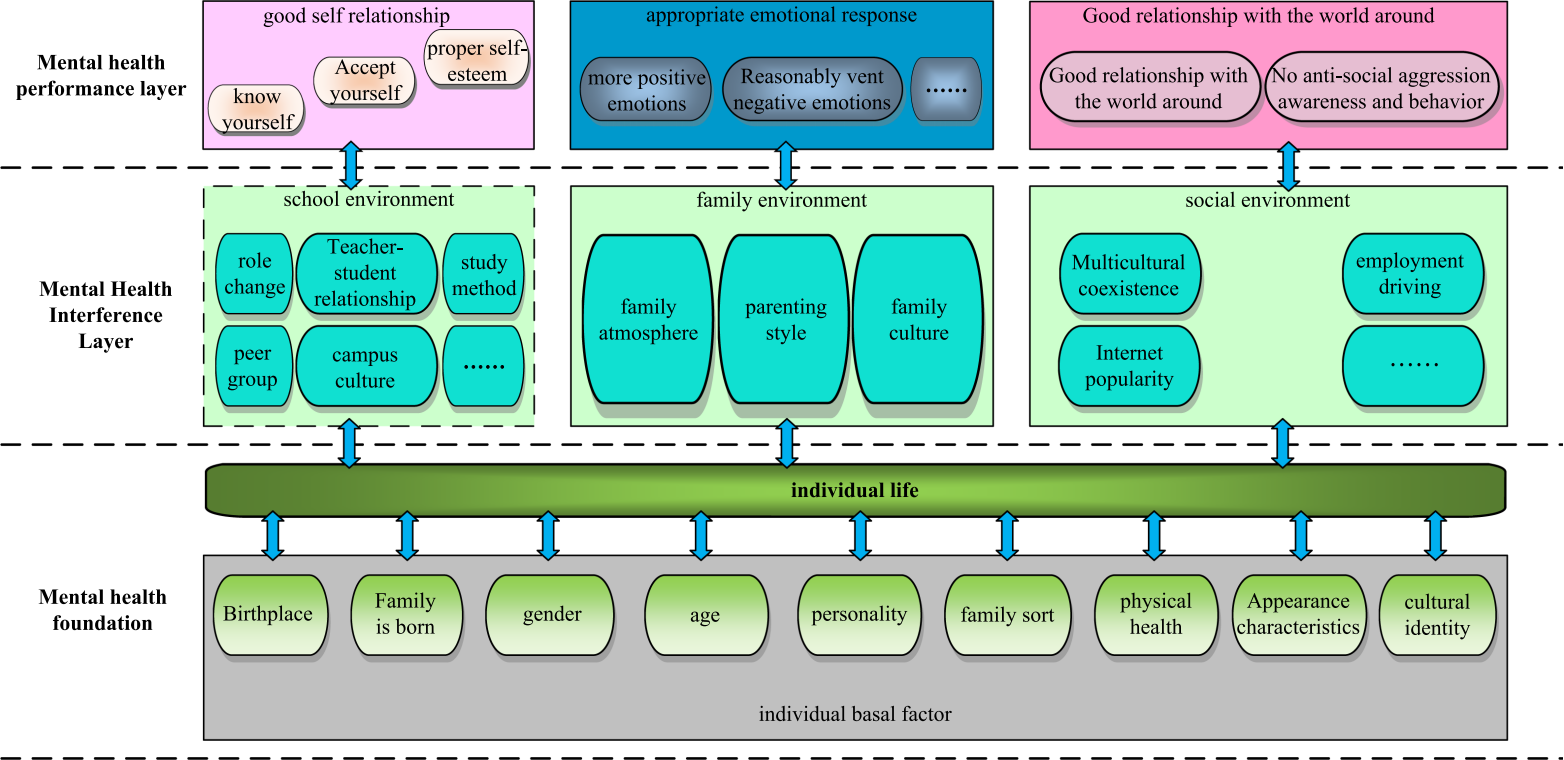
The mental model of college students.

Emotional management is a management method that recognizes and analyzes the emotions of college students, and incentive method is to use positive or negative stimulation to guide college students to establish correct concepts (Alka and Sharma, 2017; Zheng et al., 2021). The management work mode of college students that combines emotional management and motivation is a kind of educational guidance for college students’ psychology (Li et al., 2022). The psychological model of college students is shown in Figure 1.

As shown from Figure 1, the mental health of college students is divided into the basic layer, the interference layer and the performance layer from the inside out. The basic layer of mental health is determined by the basic characteristics of individual students, such as family birth, education level, gender and other characteristics that are not under their own control. The layer is the external manifestation of students’ emotions about themselves and the world around them. The main research object of this paper is the interference layer and the performance layer of college students. Analysis methods include speech emotion recognition methods, artificial neural network algorithms, and machine learning.

### Speech emotion recognition method

**Figure 2:**
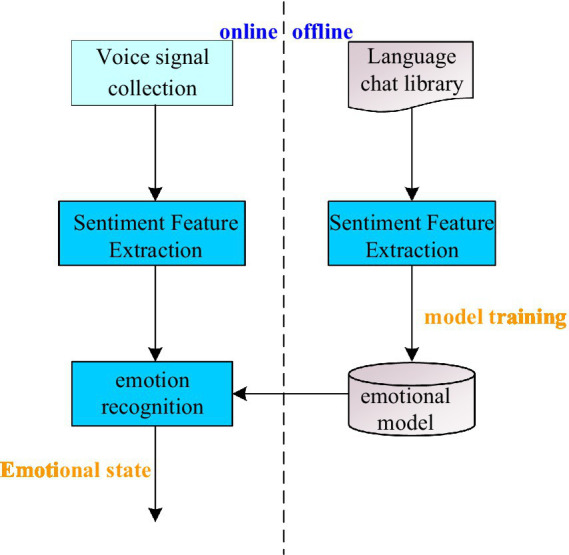
Speech emotion recognition model diagram.

Speech emotion recognition is a new direction of intelligent psychology research, which plays a great role in the management work mode of college students (Wang et al., 2017a; Jain et al., 2022). Speech emotion consists of speech emotion database, collection of speech signals, extraction of emotion features, emotion model training and recognition. The recognition model of speech emotion is shown in Figure 2.

#### Voice emotion database

The speech emotion database is the basis of speech emotion recognition, so the effect of speech emotion recognition depends on the rationality of emotion establishment in the emotion database and the richness of emotion types. The emotional speech entered in the speech emotion database must have the emotion that can express the standard state (Huang et al., 2017; Jain et al., 2020).

The establishment of the voice emotion database is generally completed by both experts in phonetics and psychology, and requires a large number of professional voice actors with emotional voice input. Currently, the commonly used voice emotional states are: surprise, sadness, neutrality, fear, angry, and happy. By filtering out unqualified voice emotions, they are finally saved in the voice emotion database.

#### Collect voice signal

##### Pre-emphasis

Since human speech will be affected by the vibration of the lips, the signal information transmitted by the speech will be weakened a lot, so it is necessary to compensate for the lost part of the speech signal, which is beneficial to the study of speech signals. Generally, speech pre-emphasis processing is adopted, and the frequency of the lost part of the speech signal is increased to the state before the loss through the filter. The pre-emphasis process of the speech signal is:


(1) $$D\left(k\right)=1-\lambda k^{-1}$$

In formula (1), λ represents the pre-emphasis parameter and k represents the speech signal frequency.

##### Framing and windowing of voice signals

The frequency of the speech signal changes with time, and it is difficult to study the overall speech signal; but in a short period of time, the speech signal can be regarded as unchanged. Therefore, the speech signal is divided into equal parts of very short speech segments, each segment is called a speech signal frame, and the speech signal frame has short-term stability, and the research on speech signals is carried out on the basis of speech frames (Wang et al., 2016; Papakostas et al., 2017). However, the speech signal frame is prone to spectrum loss. As shown, the speech signal frame needs to be windowed. The speech framed windowing is expressed as:


(2) W(x)=s(x)∗w(x)

In formula (2), W(x) represents the speech after windowing, s(x) represents the speech signal, and w(x) represents the window function.

#### Speech emotion feature parameters

##### Short-term energy

The short-term energy is the energy entrained in the speech signal frame. The short-term energy can be used to distinguish emotions. When the voice is more intense, the short-term energy is larger, and when the voice is peaceful, short-term energy is small. The short-term energy of the speech signal after framing and windowing is expressed as:


(3) $$E_{x}=\overset{N-1}{\underset{n=0}{\sum }}s_{x}^{2}\left(n\right)$$

In formula (3), AA represents the speech signal at the *x*th frame and *N* represents the number of speech frames.

Then the short-term jitter energy of the speech signal is:


(4) $$E_{d}=\frac{\frac{1}{N-1}\overset{N-1}{\underset{x-1}{\sum }}|E_{x}-E_{x+1}|}{\frac{1}{N}\overset{N}{\underset{x=1}{\sum }}E_{x}}$$

In formula (4), $E_{d}$ represents the short-term jitter energy of the speech signal.

The linear regression equation of short-term energy is expressed as:


(5) $$E_{a}=\frac{\overset{N}{\underset{x=1}{\sum }}x\cdot E_{x}-\frac{1}{N}\overset{N}{\underset{x=1}{\sum }}x\cdot \overset{N}{\underset{x=1}{\sum }}E_{x}}{\overset{N}{\underset{x=1}{\sum }}x^{2}-\frac{1}{N}\left(\overset{N}{\underset{x=0}{\sum }}x\right)}$$

In formula (5), $E_{a}$ represents the linear regression coefficient of short-term energy and *x* represents the sequence number of the speech signal frame.

The short-term energy can also judge the type of speech, distinguish unvoiced and voiced sounds, and use the short-term correlation function to extract the pitch frequency of the speech. The short-term correlation function is expressed as:


(6) $$A_{x}\left(r\right)=\overset{N-r-1}{\underset{n=0}{\sum }}s_{x}\left(n\right)s_{x}\left(n+r\right)$$

In formula (6), $A_{x}\left(r\right)$ is a short-term correlation function, *r* is an even function, and *N* represents the frame length of a speech frame.

When using short-term energy, it is usually necessary to calculate the short-term zero-crossing rate. The short-term zero-crossing rate is a way to detect the sound at both ends of the voice signal. The short-term zero-crossing rate is expressed as:


(7) $$U_{x}=\frac{1}{2}\overset{N-2}{\underset{n=0}{\sum }}\left|\mathrm{sgn}\left[s_{x}\left(n\right)\right]-\mathrm{sgn}\left[s_{x}\left(n-1\right)\right]\right|$$

In formula (7), $s_{x}$ is the speech emotion signal frame and $U_{x}$ represents the short-term zero-crossing rate, sgn stands for symbolic function.

sgn[s] is a mathematical symbolic function, which is specifically expressed as:


(8) $$\mathrm{sgn}\left[s\right]=\{\begin{array}{l} 1,\left(s\geq 0\right)\\ -1,\left(s<0\right) \end{array}$$

##### Voice emotion formant

The speech emotion formant is a frequency generated by the resonance of the speech signal, and its main function is to distinguish the final from the initial. The process of using linear prediction to estimate the speech emotion formant parameter is as follows:


(9) $$Q\left(s\right)=1-\overset{L}{\underset{i=1}{\sum }}a_{i}s^{-i},i=1,2,\cdots ,L$$

Let one of the solutions of formula (9) be $b_{i}=r_{i}e^{j\mu_{i}}$ , and find the conjugate complex root of Q(s) , then the speech emotion formant frequency is:


(10) $$G_{i}=\frac{\mu_{i}}{2\pi T}$$

In formula (10), *T* represents the period of speech signal sampling.

#### Speech emotion feature extraction

Speech emotional feature extraction is the most critical step in speech recognition. Common methods for extracting emotional features include linear prediction cepstral coefficients and Mel cepstral coefficients (Swain et al., 2018).

The mathematical representation of the Mel cepstral coefficient is:


(11) $$M\left(v\right)=2592\log_{10}\left(1+v/700\right)$$

In Equation (11), v represents the speech emotion frequency.

**Figure 3:**
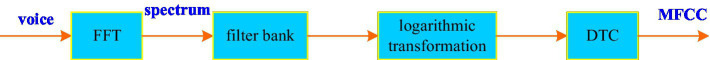
Mel cepstrum extraction process diagram of speech emotion feature.

The process of Mel cepstral coefficient extraction of speech emotion features is shown in Figure 3:

### BP neural network technology

#### Artificial neural network

An artificial neural network is a computing system that can process complex data concurrently. Since the artificial neural network imitates the working principle of the human brain, the artificial neural network has a high cognitive level, it is a good way to predict and analyze students’ emotions in the student management work mode in colleges and universities, which is also the basis for the self-learning of the artificial neural network (Wang et al., 2017b; Sachdeva and Aggarwal, 2021).

#### Neurons

**Figure 4:**
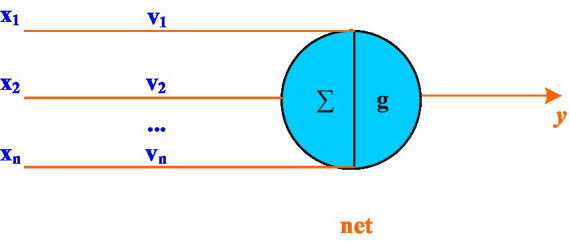
Neuron structure diagram.

Neuron is the most basic unit of neural network, and neuron is also the basic unit of neural network processing calculation and learning. The structure of neuron is shown in Figure 4.

The processing of neurons is as follows:


(12) $$\mathrm{net}=\overset{n}{\underset{j=1}{\sum }}v_{j}x_{j}$$

In formula (12), *n* represents the number of external inputs and $v_{j}$ represents the *j*th synapse of the neuron.


(13) y=g(net)

In formula (13), the function *g* represents a nonlinear function.

When the function g reaches the threshold, the output is expressed as:


(14) $$y=\mathrm{sgn}\left(\overset{n}{\underset{j=1}{\sum }}v_{j}x_{j}-g\right)$$

In formula (14), sgn represents a sign function.

Simplifying formula (14), the new expression of the threshold can be:


(15) $$\{\begin{array}{l} a=-v_{0}\\ W={\left(v_{0},v_{1},\cdots ,v_{n}\right)}^{T}\\ X={\left(1,x_{1},x_{2},\cdots ,x_{n}\right)}^{T} \end{array}$$

The output function is expressed as:


(16) $$y=\mathrm{sgn}\left(W^{T}X\right)$$

#### BP neural network structure

**Figure 5:**
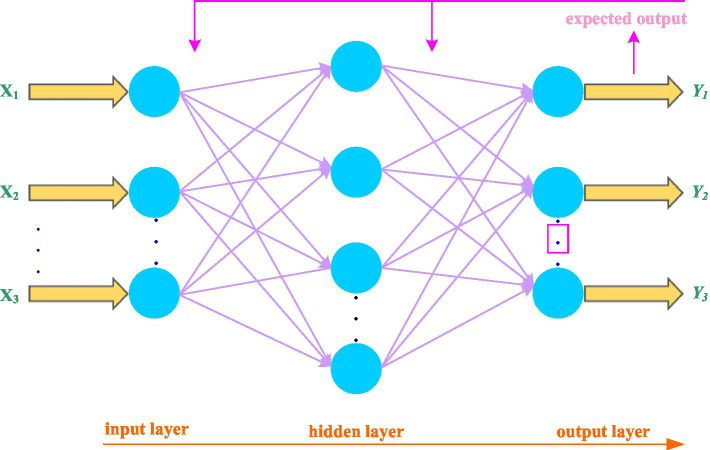
Structure diagram of BP neural network.

BP neural network is a kind of multi-layer feed-forward neural network with error back-propagation. It has very strong dynamic learning ability. It adjusts the neuron nodes according to the error of the actual output and expected output of the system, changes the structure of the BP neural network, and finally makes the actual output keep getting closer to the desired output. BP neural network is a network model with three-layer structure, and its structure is shown in Figure 5.

As shown in from Figure 5, the structure of the BP neural network from left to right is: input layer, hidden layer, and output layer (Pan et al., 2017). The error feedback of BP neural network is the core of its calculation and learning. The error can be represented by the mean variance of the actual output and expected output of the BP neural network:


(17) $$R=\frac{1}{2}\underset{n}{\sum }{\left(y_{n}^{a}-y_{n}^{b}\right)}^{2}$$

In formula (17), *R* represents the error, $y_{n}^{a}$ represents the expected output of the nth neuron, and $y_{n}^{b}$ represents the actual output of the nth neuron.

The learning process of the BP network is actually a process in which the error becomes smaller or even no error. The smallest error is expressed as:


(18) $$\nabla R=\nabla f\left(v_{1},v_{2},\cdots v_{n}\right)={\left[\frac{\partial f}{\partial v_{1}},\frac{\partial f}{\partial v_{2}},\cdots ,\frac{\partial f}{\partial v_{n}}\right]}^{T}$$

### Machine learning algorithms

**Figure 6:**
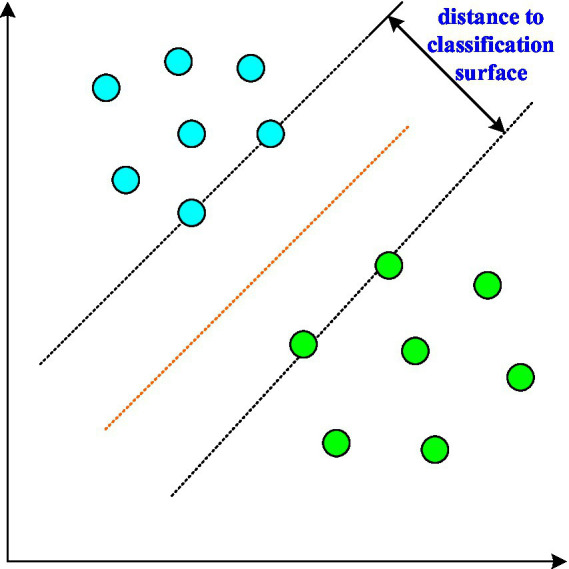
Support vector machine model diagram.

Machine learning is widely used in classification and regression prediction. In the emotional analysis of college students, machine learning can accurately classify various emotional states and provide important help for subsequent calculations (Xia et al., 2018). The most common method in machine learning is the support vector machine model, which is shown in Figure 6.

It can be seen from Figure 6 that the support vector machine has a good feature classification effect. The fundamental idea is to find the interval between two categories. The formula for the classification surface is:


(19) $$a^{T}x+b=0$$

In formula (19), $a=\left(a_{1},a_{2},\ldots ,a_{n}\right)$ is the center line of the classification surface and *b* represents the straight-line distance from the classification surface to the origin (Kyrkou et al., 2017).

The classification surface is determined by two variables *a* and *b*, which can be expressed as (a,b) , then the distance from the sample data to the classification surface is expressed as:


(20) $$d=\frac{\left|a^{T}x+b\right|}{\left|a\right|}$$

In linearly classified samples, for sample (x,y)∈R , then the following equation holds.


(21) $$\{\begin{array}{l} a^{T}x+b\geq +1,y=+1\\ a^{T}x+b\leq -1,y=-1 \end{array}$$

The distance from a support vector to the classification surface is expressed as:


(22) $$d=\frac{1}{\left|a\right|}$$

The largest interval classification surface is to maximize the result in equation (22), namely:


(23) $$\underset{a,b}{\max}\frac{1}{\left|a\right|}$$

Formula (23) can be transformed into a binary equation solving problem, and the expression after transformation is:


(24) $$\underset{a,b}{\min}\frac{1}{2}{\left|a\right|}^{2}$$

## College students’ emotional management and motivation management

### Experimental data

#### Sample data

In order to better analyze the management of college students by emotional management and incentive methods, this paper mainly compares the emotional management and incentive management of college students with the traditional college student management (Yu et al., 2021). Therefore, the selection of experimental samples is very important. Poor selection of experimental samples can easily lead to invalid experimental comparisons. And the selection of samples should cover the whole segment of the sample object as evenly as possible, in order to make the experiment with sufficient rigor.

The experimental data will select 300 students from 20 colleges and universities. Since the senior students are in the graduation season, and most of the courses are in the state of internship work, only the sample data from the first to the third grade will be considered. Among them, 100 are freshmen, 100 are sophomores, and 100 are juniors. Through a 1-month survey of the above-mentioned experimental population, the indicators that can evaluate the management ability of college students have been calculated, and the impact of college student management has been investigated as shown in Table 1.

**Table 1:** Affected situation of student management in colleges and universities.

Impact indicator	Freshman	Sophomore	Junior year
Students’ motivation to learn	83%	78%	76%
Student mental health	86%	76%	77%
Student achievement improvement rate	82%	82%	84%
Student’s cheerfulness	77%	67%	71%
Students’ motivation to participate in sports	38%	56%	60%
Attendance rate of students in class	45%	42%	39%
Average	68.5%	66.8%	67.8%

From the data in Table 1, it can be seen that the average impact of the three different college student groups of freshman, sophomore and junior college students in the six colleges and universities affected by student management is 68.5%, 66.8%, and 67.8%, respectively. All three groups of college students have been greatly affected.

#### Sample correlation

When selecting the evaluation indicators of college student management, it is necessary to carry out a correlation analysis between the impact indicators of college student management and college student management (Singh et al., 2022). Correlation analysis of samples is to prevent the failure of experiments due to improper selection of samples. Correlation analysis of samples can expand the main characteristics of the object to be studied, and it is easier to observe which indicators affect the management of college students. The results of the correlation analysis of the impact indicators in 1 are shown in Table 2.

**Table 2:** Correlation analysis table of college student management.

Number of sample groups	Impact indicator	Relevance
1	Students’ motivation to learn	0.256
2	Student mental health	0.214
3	Student achievement improvement rate	0.241
4	Student’s cheerfulness	0.229
5	Students’ motivation to participate in sports	0.024
6	Attendance rate of students in class	0.036

From the data analysis in Table 2, it can be seen that the correlation index of the student’s learning enthusiasm index is 0.256, and the index with the smallest correlation is the student’s enthusiasm index for participating in sports. The comparison of the four types is very small, so indicators five and six are excluded, and the first four impact indicators are marked as impact indicators with a high degree of correlation.

#### Validity of the sample

In order to test whether the first four index data in Table 2 are effective for the comparison experiment of emotional management and incentive college student management and traditional college student management, the experiment will be tested by k-fold cross-validation. Due to the data dimension of this experiment, it is not large, so the 5-fold cross-validation method is used (Sjp and Kcc, 2019). The experiment selects 300 college students as the verification data, of which 240 college students are the test set, and the remaining 60 students are the test set. The experiment will be carried out five times, so that each student can become the test data and the test data, and the final result is the average of five validation results, and the validity analysis results of 5-fold cross-validation are shown in Table 3.

**Table 3:** Effectiveness analysis result table.

Impact indicator	Emotional management and encouragement of college students’ management	Traditional college student management
Students’ motivation to learn	86.4%	73.2%
Student mental health	78.8%	76.8%
Student achievement improvement rate	84.4%	82.6%
Student’s cheerfulness	92.8%	78.2%
Average	85.6%	77.7%

The data in Table 3 shows that the highest effectiveness index in the management of college students based on emotional management and motivation is the degree of cheerfulness of students, and the average effectiveness of the above four influencing indicators is 85.6%; the effectiveness of traditional college student management is the highest indicator is the student’s performance improvement rate, and the average validity of the impact indicator is 77.7%. Since the above four indicators have high effectiveness in the two modes of college student management, we can conduct comparative experiments on the above four aspects of emotional management and incentive college student management and traditional college student management.

### Comparison experiment between emotional management and motivational college student management and traditional college student management work mode

Emotional management and incentive management of college students make corresponding management measures by analyzing students’ emotions and guide students in an incentive way. Traditional college student management still adopts institutional management. The experiment will compare the students’ enthusiasm for learning, students’ mental health, students’ achievement improvement rate, and students’ cheerfulness in these two work modes of college student management (Washio, 2018).

#### Comparative experiment of students’ learning enthusiasm

The most direct criterion to reflect the quality of college students’ management work mode is the students’ learning status, and the students’ learning status is affected by the students’ learning enthusiasm, and the students’ learning enthusiasm also reflects the students’ psychological activity. In order to make the experiment more convincing, the experiment was conducted with 100 students in each of the freshman, sophomore, and junior grades for a period of 6 months. The two types of college student management work models accounted for half of the experimental students. Record students’ learning enthusiasm once a month. In addition to this comparative experiment.

**Figure 7:**
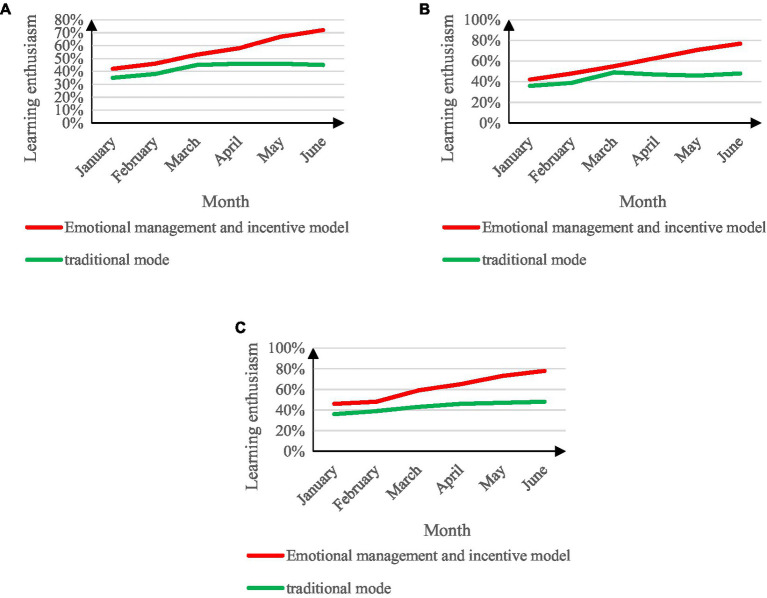
Comparison of students’ enthusiasm for learning. **(A)** The enthusiasm of the first-year students to study. **(B)** The enthusiasm of the second-year students to study. **(C)** Enthusiasm for junior students to study.

Except for the different modes of college student management work adopted, others are the same. The comparison results of students’ learning enthusiasm in the two college student management work modes are shown in Figure 7.

From the analysis in Figure 7, it can be seen that the enthusiasm of the three grades varies slightly between months, but the difference is small. During the 6 months of the experiment, the general trend of the development curve of the students’ learning enthusiasm is the same. In the work mode of emotional management and motivation of college student management, students’ learning enthusiasm has been on the rise, and the learning enthusiasm in the best period is 78%. In the traditional management mode of college students, the enthusiasm of students occupies an important position. The monthly growth rate was stable, and the growth rate was slow in the next 3 months, and even declined. The learning enthusiasm in the best period was 48%.

#### Comparison experiment of students’ mental health degree

**Figure 8:**
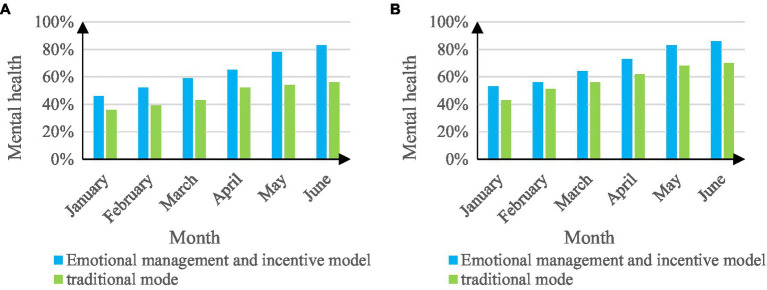
Comparison chart of students’ mental health. **(A)** Mental health of female students. **(B)** Mental health of male students.

The psychological health of students is an important criterion to reflect the psychological state of students. The experiment conducted statistics on the psychological health of students, including 160 males and 140 females. The experiment was carried out for 6 months, and data statistics were carried out once a month. The reason for distinguishing between males and females for the experiment is that there are differences in the psychological conditions of boys and girls. The results of the comparison of the psychological health of students in the two college student management work modes are shown in Figure 8.

From Figure 8A, the college student management work mode of emotional management and motivation is better than the traditional work mode in the mental health of students in the early stage of the experiment, and with the progress of the experiment, the emotional management and motivation mode improves. The greater the increase in the level of mental health of students. In figure (B), the overall trend is similar to that in figure (A), and it also shows that the mental health of students in the emotional management and motivation model is better than that in the traditional model. Comparing the two figures (A, B), it can be seen that the mental health of boys is generally better than that of girls.

#### Comparison experiment of students’ achievement improvement rate

**Figure 9:**
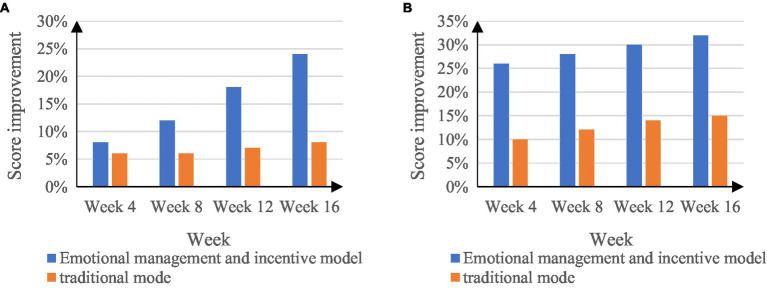
Comparison of student achievement improvement rates. **(A)** The rate of improvement for students in the previous semester. **(B)** The rate of improvement for students in the next semester.

The student management work mode in colleges and universities directly reflects the students’ grades. The level of student performance is determined by the basis of students’ learning, but a large part of the improvement rate of grades is caused by the work mode of college students’ management work. To this end, an experiment was conducted to compare the rate of achievement improvement among college students. The experimental period was the entire academic year of the students, each semester had 16 weeks, and a survey of the rate of improvement in students’ achievement was conducted every 4 weeks. Figure 9 shows the comparison results of the improvement rate of students in the two colleges and universities student management work modes.

In Figure 9, the student’s grade improvement rate in the last semester under the emotional management and motivation mode of college student management is 24%, and the student’s grade improvement rate under the traditional college student management mode is 8%; in the next semester, students’ grades will improve rates by 32% and 15%, respectively.

#### Comparison experiment of students’ cheerfulness degree

**Figure 10:**
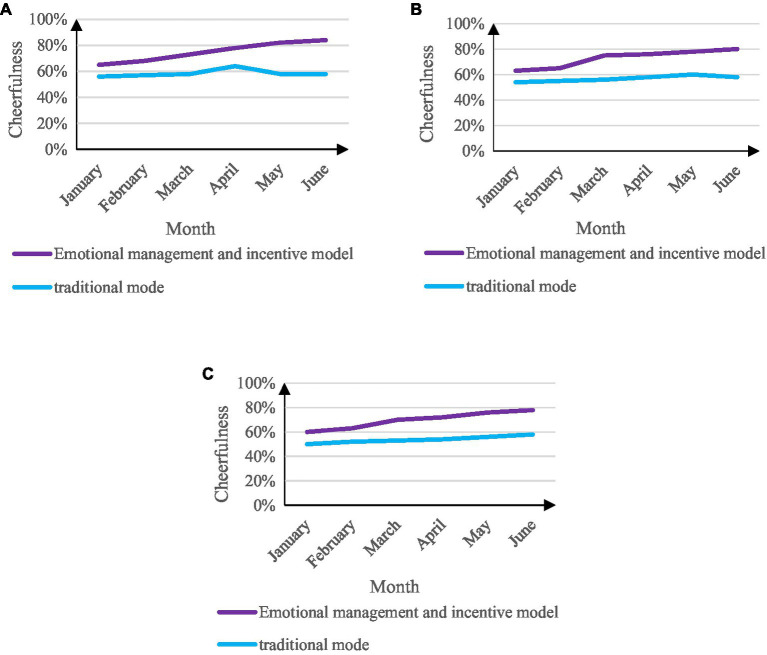
Comparison of students’ cheerfulness. **(A)** The degree of cheerfulness of freshman students. **(B)** The degree of cheerfulness of sophomore students. **(C)** The degree of cheerfulness of the third-year students.

Another criterion for judging the student management work mode in colleges and universities is the degree of cheerfulness of the students. The experiment is carried out on all students in colleges and universities. Except for the senior students, the experiment is carried out for 6 months, and the data on the cheerfulness of the students are investigated once a month shown in Figure 10.

From the data analysis in Figure 10, it can be seen that the students’ personality is much better than the traditional college student management work mode under the emotional management and motivation mode of college student management work. And with the progress of the experiment, the cheerfulness of students in the college student management work model of emotional management and motivation has increased rapidly. The best period is 84% in the sixth month of the freshman experiment. The cheerfulness degree of the students in the mode increased slowly, and even showed a slight downward trend, and the best period of cheerfulness was 58%.

### Experimental

Through a multi-faceted comparison of the two college student management work modes, the experimental results show that under the college student management work mode based on emotional management and incentives, students’ learning enthusiasm, students’ mental health, students’ achievement improvement rate, and students’ cheerful disposition, the four aspects of the degree are better than the traditional college student management work mode. The average data comparison of the two methods of college student management work mode is shown in Table 4.

**Table 4:** Comparison of the average data of the two modes of college student management work.

Compare items	Emotional management and motivation of college students’ management work mode	The traditional management mode of college students
Students’ motivation to learn	59.1%	43.3%
Student mental health	66.5%	52.5%
Student achievement improvement rate	22.3%	9.8%
Student’s cheerfulness	72.6%	56.4%

## Discussion

There are many defects in the traditional student management work model in colleges and universities, and students cannot develop well. With the advancement of sentiment analysis technology and the development of motivation theory, the method of emotional management and motivation is combined with the traditional management mode of college students, and through psychological guidance and emotional analysis of students, to better manage college students (Ravi and Rajagopalan, 2020). By implementing emotional management and incentive methods for college students, it can effectively improve students’ enthusiasm for learning, students’ mental health, and students’ performance, get rid of psychological problems, and guide college students to be positive from the psychological aspect.

## Conclusion

The experimental results show that: (1) Compared with traditional methods, emotional management and incentive methods can make college students improve in learning. The students’ learning enthusiasm based on emotional management and motivation in college student management work mode and traditional college student management work mode are 59.1% and 43.3%, respectively. In terms of student achievement, the student achievement improvement rates of the two college student management work models were 22.3% and 9.8%, respectively. (2) Compared with traditional methods, emotional management and incentive methods can improve the psychological aspects of college students. In the 6-month experiment, the emotional management and motivation methods are 14% higher than the traditional methods in the students’ mental health and 16.2% in the students’ cheerfulness. All in all, the management work mode of college students based on emotional management and incentives is more effective than the traditional management mode of college students. However, the current college student management work mode based on emotional management and incentives is still realized by technologies such as voice emotion technology. In the future, a variety of emotion recognition technologies can be combined with college student management work modes to improve the accuracy of emotion recognition is the reverse of future research.

## Data availability statement

The original contributions presented in the study are included in the article/supplementary material, further inquiries can be directed to the corresponding author.

## Ethics statement

Ethical approval for this study and written informed consent from the participants of the study were not required in accordance with local legislation and national guidelines.

## Author contributions

The author confirms being the sole contributor of this work and has approved it for publication.

## Conflict of interest

The author declares that the research was conducted in the absence of any commercial or financial relationships that could be construed as a potential conflict of interest.

## Publisher’s note

All claims expressed in this article are solely those of the authors and do not necessarily represent those of their affiliated organizations, or those of the publisher, the editors and the reviewers. Any product that may be evaluated in this article, or claim that may be made by its manufacturer, is not guaranteed or endorsed by the publisher.
